# Persistence and stability of sublingual varices over time and their connection to underlying factors: an 8 year follow up study

**DOI:** 10.1186/s12903-022-02379-9

**Published:** 2022-08-11

**Authors:** Håkan Bergh, Clovis Kastberg, Margit Albrektson, Lennart Hedström

**Affiliations:** 1 School of Public Health and Community Medicine, Institute of Medicine, University of Gothenburg, Gothenburg, Sweden; 2 Department of Research and Development Unit, Hospital Varberg, Region Halland, Halmstad, Sweden; 3 Tre Tandläkare, Varberg, Sweden; 4 Public Dental Services, Västra Vall, Varberg, Sweden; 5 Hajvägen 37, 432 74 Varberg, Sweden

**Keywords:** Sublingual varices, Persistence, Cardiovascular risk factors

## Abstract

**Objective:**

To investigate whether sublingual varices are constant or inconstant over time and whether this is connected to background variables, cardiovascular risk factors or disease.

**Design:**

This longitudinal observational study was performed between 2010 and 2020 at the Public Dental Services Västra Vall, Varberg, Sweden. The study was based on 431 patients included in a previous study in which the relationship between sublingual varices and hypertension was examined. In connection to the annual dental examination, 281 patients were included in the follow-up study. They completed a questionnaire about background and health factors and diseases. Length and weight were measured. Two intraoral photos were taken with a digital camera. Two dentists independent of each other graded all photographs as none/few visible sublingual varices (nSV) or medium/severe sublingual varices (SV).

**Results:**

The prevalence of SV was 25.6% at baseline and 30.6% at follow-up. At the follow up, a total of 76.5% had maintained their sublingual vascular status. Of those with nSV at baseline (n209), 80.9% still had nSV, and 19.1% had developed SV during the 8-year follow-up period. Of those 72 participants who had SV at baseline, 46 (63.9%) were unchanged at follow-up, and 26 (36.1%) were classified as nSV. Those who had developed SV at follow-up had a higher mean age (*p* = 0.003) and a higher prevalence of cardiovascular disease (CVD), 13.2% versus 3.0% (*p* = 0.021). This association with CVD did not persist after an adjustment for sex and age (OR 3.2, 95% CI 0.81–12.46). They exhibited more hypertension (35.0% vs. 22.5%) and diabetes type 2 (7.5% vs. 3.0%), but with no significant difference.

**Conclusions:**

This study revealed that 76.5% of the participants had an unchanged status regarding sublingual varices during an 8-year period and that the development from nSV to SV was associated with advanced age.

## Background

Sublingual varices (SV) is a common vascular lesion with different denominations, such as caviar tongue, sublingual/lingual varicosities, phlebectasia linguae, oral varix, and vascular malformations. These dilatations are mostly situated at the undersurface of the tongue, are asymptomatic, and there is no need for treatment [1]. The prevalence of SV is approximately 23–35%, depending on which definition of SV is used and the age of the study population [2–5]*.* The origin of SV development is unknown. It could be an ageing process, as SV increases with age. The association between sublingual varices and other conditions such as varicose veins and liver cirrhosis has been studied previously [6, 7]. More recently, research has shown a connection with smoking [2, 8–10], hypertension [3, 8–12], diabetes mellitus type 2 [8, 9, 12] cardiovascular disease (CVD) [2, 4, 5], denture wearing [4].

As there is a correlation between SV and hypertension, SV could be an indicator of risk for hypertension [3]. This indicator could be used in dental settings to identify patients with SV and further examination of hypertension could be recommended in a medical setting. It is therefore essential to determine if SV is a temporary or persistent phenomenon over time and if this is linked to general and/or medical factors. There are no previous studies addressing this topic. The purpose of this study was to investigate whether SVs are constant or inconstant over time and whether this is connected to background variables, cardiovascular risk factors or disease.

## Methods

### Design and setting

This longitudinal observational study was performed between 2010 and 2020 at the Public Dental Services Västra Vall, Varberg, Sweden.

### Study population

Patient selection was based on patient material that was included in a previous study performed between May 2010 and February 2013, in which the relationship between SV and high blood pressure was examined among 431 consecutive patients ≥ 40 years of age [3]. Exclusion criteria were pregnancy, atrial fibrillation or renal disease. In association with the patients’ annual dental examination, they received a written description of the follow-up study, and when they arrived at the planned visit, they were asked about participation. Those who accepted participation were consecutively enrolled. After 281 patients were included in the follow-up study (May 2018 to March 2020), recruitment was terminated prematurely due to the pandemic, and the remaining 150 subjects had their annual visit postponed. The patients received both written and verbal information about the study and signed an informed consent. The study was approved by the Regional Research Ethics Committee at the University of Lund (EPN 2018/60) and in accordance with the Helsinki Declaration.

### Procedure, data collection and processing

**Fig. 1:**
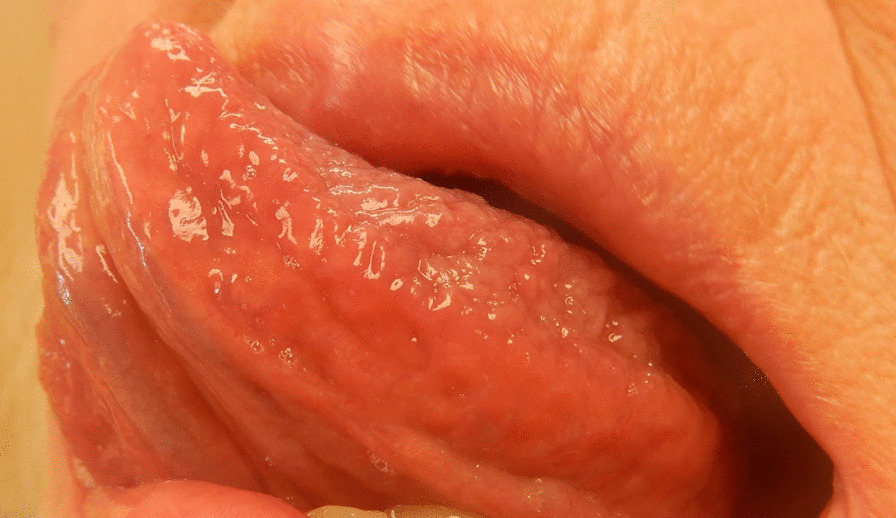
None/few sublingual varices (grade 0). Written consent obtained from patient

**Fig. 2:**
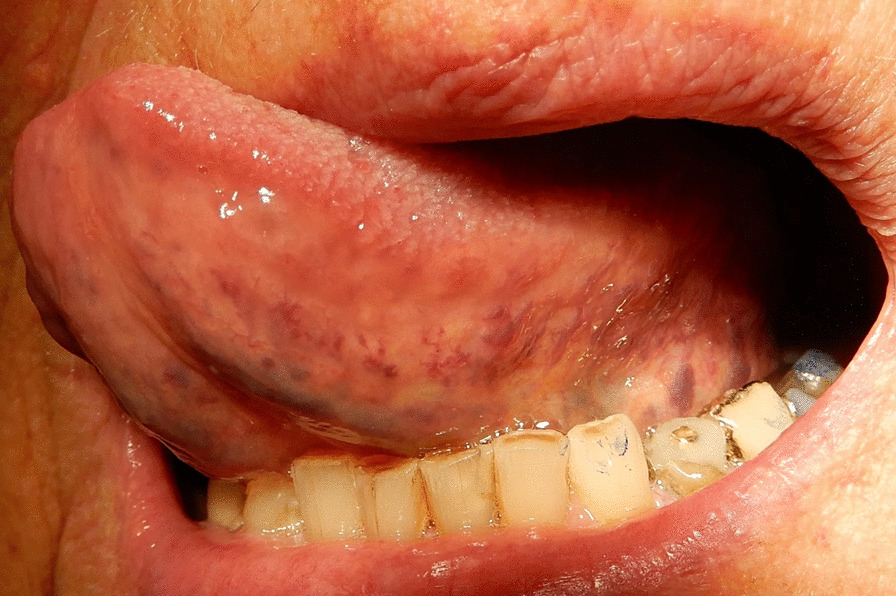
Moderate/severe sublingual varices (grade 1). Written consent obtained from patient

The patients who arrived for their annual dental check-up completed a questionnaire about background and health factors and diseases. Two intraoral photos were taken with a digital camera (Nikon 35 × 4 k Coolpix) on each side of the undersurface of the tongue. After the oral examination, the participants’ height (cm) and weight (kg) were measured in a standing position without shoes. The following data were collected: age (in years); gender (male/female); tobacco habits (do you smoke/use snuff) with answer options: yes daily, yes sometimes, no; diseases: have you been diagnosed by a physician with high blood pressure, type 2 diabetes (DM2), high blood lipids, myocardial infarction, angina pectoris, atrial fibrillation, stroke, lower limb varices, or dementia with answers options: yes, no, do not know. The smoking variable was dichotomized into non-smoking/smoking (daily or sometimes smoking). Snuffing was dichotomized in the same way. Diagnoses were dichotomized into yes/no where the do not know option was included in no. Myocardial infarction, angina and stroke were merged into a compound variable: CVD. The photographs were assessed by three general practitioner dentists (LH, KH, POC) blinded to the patient's questionnaire and measurement data and to each other's assessment. One dentist (LH) has assessed SV since 2003, and he has trained the other two (KH, POC) in assessments. All photographs were graded as none/few visible sublingual varices (grade 0 = nSV) (Fig. 1) or medium/severe sublingual varices (grade 1 = SV) (Fig. 2). Consensus was reached in cases in which the initial assessment differed between observers.

### Statistical analysis

For group comparisons of ordinal data, the Chi-squared test or Fisher’s exact test was used. Student’s t test was used in group comparisons of numerical data. The influence of independent variables on the dependent variable (SV/no SV at follow-up) was calculated using logistic regression analysis (enter model), adjusted for sex and age. Data were classified according to the following: nSV/SV (0/1), male/female (0/1), no CVD/CVD disease (0/1). The agreement between the two independent assessors of the photographs on the sublingual varicose veins was calculated with Cohen’s kappa coefficient. For calculations, SPSS version 27.0 was used and the significance level was set at *p* < 0.05.

## Results

Data from 281 participants were included in the analysis. Mean age at follow-up was 63.1 years and the majority of subjects were women (54%). During the 8 years between baseline and follow-up, the prevalence of hypertension, DM2, hyperlipidaemia and CVD increased (Table 1). The prevalence of SV was 25.6% at baseline and 30.6% at follow-up. At the follow-up, 76.5% of subjects maintained their sublingual vascular status, while a status alteration had occurred among 23.5% of subjects. Of the initial 209 patients with nSV, 80.9% had an unchanged SV status, and 19.1% had developed SV during the 8 years follow-up period. Of those 72 participants who had SV at baseline, 46 (63.9%) were unchanged at follow-up, and 26 (36.1%) were classified as nSV (Table 2).

**Table 1:** Description of the study population’s background variables and cardiovascular riskfactors and disease at baseline and follow up (n281)

	Baseline	Follow up
Male/female, n/n (%/%)	128/153	128/153
	(45.6/54.4)	(45.6/54.4)
Mean age, year (SD)	55.2 (9.9)	63.1 (9.9)
BMI, kg/m^2^ (SD)	25.7 (4.1)	26.2 (4.4)
Smoking, n (%)	25 (8.9)	21 (7.5)
Snuff user, n (%)	28 (10.0)	29 (10.4)
Hypertension, n (%)	62 (22.1)	90 (32.0)
Diabetes type 2, n (%)	14 (5.0)	20 (7.1)
Hyperlipidemia, n (%)	23 (8.2)	38 (14.0)
Cardiovascular disease, n (%)	10 (3.6)	21 (7.6)
Sublingual varices, n (%)	72 (25.6)	86 (30.6)

**Table 2:** The presence of sublingual varices (SV) and no sublingual varices (nSV) in the study population at baseline and follow up (n281)

	Follow up		
	nSV	SV	
*Baseline*			
nSV n (%)	169 (80.9)	40 (19.1)	209 (100)
SV n (%)	26 (36.1)	46 (63.9)	72 (100)
Total n (%)	195 (69.4)	86 (30.6)	281 (100)

Table 3 presents baseline and follow-up data of the group with nSV at baseline (n209). At follow-up, the group was divided based on sublingual status at follow-up and compared with regard to the presence of risk factors and diseases. Those who had developed SV at follow-up had a higher mean age (*p* = 0.003) and a higher prevalence of CVD, 13.2% versus 3.0% (*p* = 0.021). They reported more hypertension (35.0% vs. 22.5%) and DM2 (7.5% vs. 3.0%), however, with no significant difference. The significant association between developed SV at follow-up and CVD did not remain in the logistic regression analysis, adjusted for sex and age (OR 3.2, 95% CI 0.81–12.46).

**Table 3:** Background variables, cardiovascular risk factors and disease among subjects with no sublingual varices (nSV) at baseline (n209)

	nSV n209	nSV n169	SV n40	*p* value
	Baseline	Follow up	Follow up	
Male/female, n/n (%/%)	86/123 (41.1/58.9)	68/101 (40.2/59.8)	18/22 (45.0/55.0)	0.582**
Mean age, years (SD)	53.0 (8.6)	59.9 (8.2)	64.3 (9.2)	0.003*
BMI, kg/m^2^ (SD)	25.2 (4.0)	26.0 (4.5)	25.8 (3.9)	0.849*
Smoking, n (%)	17 (8.1)	11 (6.5)	3 (7.5)	0.734***
Snuff user, n (%)	13 (6.2)	13 (7.7)	4 (10.0)	0.747***
Hyperlipidaemia, n (%)	14 (6.7)	17 (10.4)	3 (7.7)	0.771***
Hypertension, n (%)	36 (17.2)	38 (22.5)	14 (35.0)	0.100**
Diabetes type 2, n (%)	8 (3.8)	5 (3.0)	3 (7.5)	0.183***
Cardiovascular disease, n (%)	4 (1.9)	5 (3.0)	5 (13.2)	0.021***

The group was divided by the presence or absence of sublingual varices at follow-up (SV/nSV). The variable distributions at follow-up were compared between the two groups

^*^Student’s t test

^**^Pearson Chi-square test

^***^Fisher’s exact test

Of the 72 individuals who had SV at the baseline examination, 63.9% had unchanged sublingual status at follow-up, while 36.1% had normalized sublingual veins. Men dominated the group (65.2%) that had retained the SV at follow-up, and a higher mean age distinguishes this group from those who had normalized blood vessels at follow-up, 72.5 versus 64.7 years (*p* = 0.002) (Table 4). Those who retained their SV at follow-up reported a higher prevalence of CVD and DM2, 19.6% versus 7.7% and 19.6% versus 11.5%, respectively, but the difference was not significant. The interobserver agreement regarding photographs of the tongue, expressed as Cohen’s kappa coefficient, was 0.73.

**Table 4:** Background variables, cardiovascular risk factors and disease among subjects with sublingual varices (SV) at baseline (n72)

	SV n72	nSV n26	SV n46	*p* value
	Baseline	Follow up	Follow up	
Male/female, n/n (%/%)	42/30 (41.7/58.3)	12/14 (46.2/53.8)	30/16 (65.2/34.8)	0.115**
Mean age, years (SD)	61.5 (10.6)	64.7 (9.3)	72.5 (10.5)	0.002*
BMI, kg/m^2^ (SD)	27.1 (3.8)	27.7 (3.7)	26.7 (4.5)	0.374*
Smoking, n (%)	8 (11.1)	2 (7.7)	5 (11.1)	1.000***
Snuff user, n (%)	15 (20.8)	7 (26.9)	5 (10.9)	0.104***
Hyperlipidaemia, n (%)	9 (12.5)	6 (23.1)	12 (27.9)	0.658**
Hypertension, n (%)	26 (36.1)	14 (53.8)	24 (52.2)	0.891**
Diabetes type 2, n (%)	6 (8.3)	3 (11.5)	9 (19.6)	0.517***
Cardiovascular disease, n (%)	6 (8.3)	2 (7.7)	9 (19.6)	0.307***

The group was divided by the presence or absence of sublingual varices at follow-up (SV/nSV). The variable distributions at follow-up were compared between the two groups

^*^Student’s t test

^**^Pearson Chi-square test

^***^Fisher’s exact test

## Discussion

The main finding in this survey is that 76.5% of subjects had an unchanged SV status during an 8 year follow-up period, and among those with newly developed SV during the study period, CVD was more common. Since no one has previously studied SV stability over time, there are no comparative results to relate to these findings. The prevalence of SV increased from 25.6% at baseline to 30.6% at follow-up; during this period, the participants became 8 years older. It is well known that the prevalence of SV increases with increasing age [2–5]. The newly developed SV from baseline to follow-up are mainly explained by the increasing age among the participants. CVD was more common both among subjects with new SV (13.2%) and among those with remaining SV (19.6%) at follow-up compared with those without SV (3.0% and 7.7%). This relationship is weak due to a small study population and disappears when age is adjusted. However, there is a remarkably high prevalence of CVD among subjects with SV. The results also show that this group has a relatively high incidence of both hypertension and DM2. A connection between SV and hypertension has been shown in earlier studies [3, 8, 9, 11, 12]. Previous studies have found a connection between SV and CVD [2, 4, 5]. In the aforementioned studies, hypertension was included among CVD. In the current study, heart attack, angina and stroke, but not hypertension, were included in the variable CVD. The connection between SV and hypertension and the possible connection between SV and CVD and DM2, respectively, are particularly interesting in light of the fact that the pathophysiological explanation for why SV occurs is unknown. If the emergence of SV was a purely age-related phenomenon, then there should be no association with hypertension and smoking, which has been shown in previous studies. A connection between SV and hypertension cannot be explained by a high intravascular pressure because an increased arterial blood pressure cannot be transmitted directly to veins via a capillary bed. In addition, veins above the heart level, such as the sublingual veins, have negative pressure. Hypertension, DM2 and smoking are well-established risk factors for CVD. A recently published study found an association between SV and; hypertension, high systolic blood pressure, DM2, high fasting plasma glucose values, dyslipidemia and abdominal obesity [8]. The higher incidence of CVD in subjects with SV is exciting even if there is a weak connection. These results need to be verified in larger studies.

One limitation of the study is the unvalidated questionnaire. Since the simple formulation of the questions (“have you been diagnosed by a physician with DM2, myocardial infarction, etc.?”) and with few alternative answers (yes/no/do not know), the risk of misunderstandings is small. The plan was to include a greater number of patients, but due to the pandemic during 2020, inclusion ended prematurely, resulting in low numbers of some risk factors and diseases. The assessment of the sublingual veins is subjective, but the interobserver agreement of 0.73 indicates a fairly good agreement. The above weaknesses must be taken into account when interpreting the results. Although this is a small study, it is the only study that has studied SV over time.

## Conclusion

The study revealed that 76.5% of the participants had unchanged sublingual veins status, and 14% had developed SV during an 8-year period. Subjects who had retained their SV and newly emerged SV had a higher average age and reported more CVD, but not statistically significant.

## Data Availability

The datasets generated and analysed during the current study are not publicly available due to the sensitivity of the data collected. Some data may be available from the corresponding author [HB] upon reasonable request. The data is in Swedish.
